# 2,3-Dimethyl-1*H*-imidazol-3-ium chloride

**DOI:** 10.1107/S2414314620006604

**Published:** 2020-05-19

**Authors:** Grace Anderson, Arsalan Mirjafari, Matthias Zeller, Patrick C. Hillesheim

**Affiliations:** a Department of Chemistry and Physics, Florida Gulf Coast University, 10501 FGCU Blvd. South, Fort Myers, FL, 33965, USA; b Purdue University, Department of Chemistry, 560 Oval Drive, West Lafayette, Indiana, 47907, USA; c Ave Maria University, Department of Chemistry and Physics, 5050 Ave Maria Blvd, Ave Maria FL, 34142, USA; Howard University, USA

**Keywords:** crystal structure, imidazolium, ionic, hydrogen bonding

## Abstract

The title salt, C_5_H_9_N_2_^+^·Cl^−^, exhibits multiple hydrogen-bonding inter­actions between the cationic imidazole moiety and the chloride anion. The protonated aromatic nitro­gen moiety displays the shortest hydrogen-bonding inter­actions while weaker hydrogen bonding is observed between the aromatic H atoms and the chloride anion. The crystal studied was refined as a two-component inversion twin with a twin ratio of 0.71 (5) to 0.29 (5).

## Abstract 2

The title compound crystallizes in an ortho­rhom­bic unit cell with a single cation–anion pair in the asymmetric unit. Hydrogen bonding appears to be the dominant inter­molecular force between the individual ions, forming extended networks.

## Graphical abstract

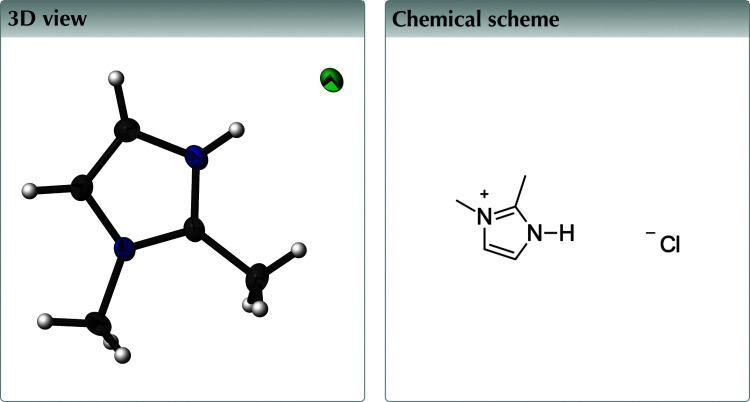

## Structure description

**Figure 1:**
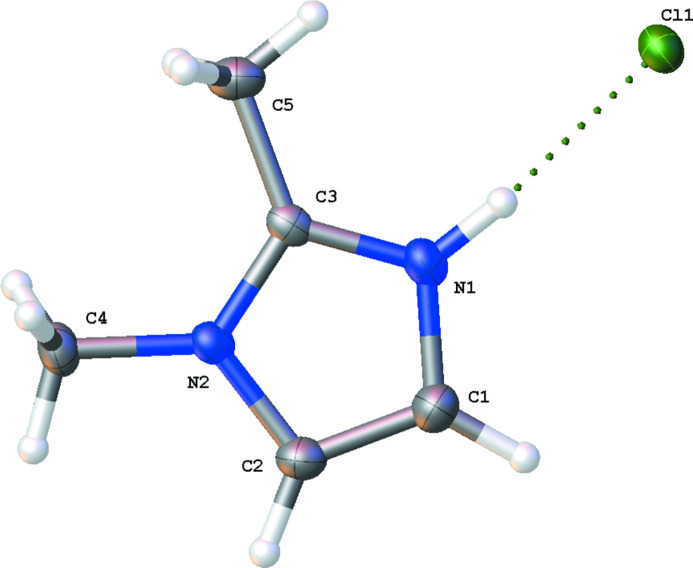
The title compound shown with 50% probability ellipsoids. Carbon (grey), hydrogen (white), nitro­gen (blue), chlorine (green).

**Figure 2:**
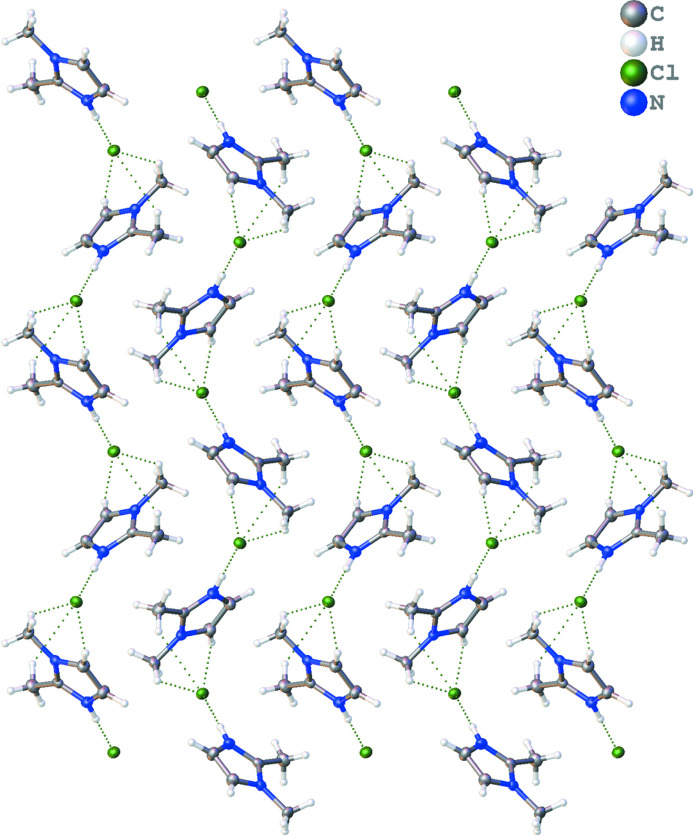
Packing diagram of the title compound showing a zigzag network of ion pairs held together through hydrogen bonds.

The title structure, 2,3-dimethyl-1*H*-imidazol-3-ium chloride (Fig. 1 ▸), crystallizes in the *P*2_1_2_1_2_1_ ortho­rhom­bic space group with a single cation–anion pair in the asymmetric unit. The acidic hydrogen, H1, exhibits a strong hydrogen bond to the chloride anion with a distance of 2.122 (19) Å. Longer hydrogen bonds between the chloride anion and H atoms on both methyl groups on the imidazolium ring as well as to the aromatic H atoms on adjacent cations form the dominant inter­molecular inter­actions in the overall network (see Table 1 ▸). The positioning of the cations, likely to facilitate hydrogen bonding, also precludes any possible long-distance π–π inter­actions given the canted angles of the rings with respect to each other (see Fig. 2 ▸).

## Synthesis and crystallization

1,2-Di­methyl­imidazole (0.2568 g, 2.662 mmol) and trityl chloride (0.7439 g, 2.668 mmol) were dissolved in separate 50 mL beakers with toluene. The reactants were then combined in a single-necked 100 mL round-bottom flask equipped with a magnetic stir bar and left to stir for 2 d at room temperature. The solvent was removed under vacuum leaving a white solid residue. This solid was washed twice with tetra­hydro­furan and recovered *via* vacuum filtration. Crystals were grown at room temperature by vapor diffusion with aceto­nitrile as the solvent and tetra­hydro­furan as the anti-solvent. Colorless crystals of the hydrolyzed byproduct (2,3-dimethyl-1*H*-imidazol-3-ium chloride) were observed within one week.

## Refinement

Crystal data, data collection and structure refinement details are summarized in Table 2 ▸. The crystal studied was refined as a two-component inversion twin with a twin ratio of 0.71 (5) to 0.29 (5).

## Supplementary Material

Crystal structure: contains datablock(s) I. DOI: 10.1107/S2414314620006604/bv4030sup1.cif

Structure factors: contains datablock(s) I. DOI: 10.1107/S2414314620006604/bv4030Isup2.hkl

Supporting information file. DOI: 10.1107/S2414314620006604/bv4030Isup3.cml

CCDC reference: 2004253

Additional supporting information:  crystallographic information; 3D view; checkCIF report

## Figures and Tables

**Table 1:** Hydrogen-bond geometry (Å, °)

*D*—H⋯*A*	*D*—H	H⋯*A*	*D*⋯*A*	*D*—H⋯*A*
N1—H1⋯Cl1	0.923 (19)	2.122 (19)	3.0396 (10)	172.3 (17)
C2—H2⋯Cl1^i^	0.955 (17)	2.695 (17)	3.6084 (11)	160.2 (13)
C4—H4*C*⋯Cl1^ii^	1.005 (19)	2.85 (2)	3.6601 (12)	138.3 (14)
C5—H5*A*⋯Cl1^ii^	0.94 (2)	2.887 (19)	3.6415 (13)	138.0 (14)

Symmetry codes: (i) 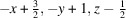 ; (ii) 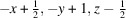 .

**Table 2:** Experimental details

Crystal data	
Chemical formula	C_5_H_9_N_2_^+^·Cl^−^
*M*_r_	132.59
Crystal system, space group	Orthorhombic, *P*2_1_2_1_2_1_
Temperature (K)	150
*a*, *b*, *c* (Å)	6.3076 (4), 9.5490 (6), 11.3951 (8)
*V* (Å^3^)	686.34 (8)
*Z*	4
Radiation type	Mo *K*α
μ (mm^−1^)	0.46
Crystal size (mm)	0.53 × 0.49 × 0.42

Data collection	
Diffractometer	Bruker AXS D8 Quest CMOS
Absorption correction	Multi-scan (*SADABS*; Krause *et al.*, 2015 ▸)
*T*_min_, *T*_max_	0.713, 0.747
No. of measured, independent and observed [*I* > 2σ(*I*)] reflections	13899, 2602, 2499
*R*_int_	0.029
(sin θ/λ)_max_ (Å^−1^)	0.768

Refinement	
*R*[*F* ^2^ > 2σ(*F* ^2^)], *wR*(*F* ^2^), *S*	0.019, 0.050, 1.11
No. of reflections	2602
No. of parameters	111
H-atom treatment	All H-atom parameters refined
Δρ_max_, Δρ_min_ (e Å^−3^)	0.21, −0.15
Absolute structure	Refined as an inversion twin
Absolute structure parameter	0.29 (5)

Computer programs: *APEX3* (Bruker, 2018 ▸), *SAINT* (Bruker, 2018 ▸), *SHELXS97* (Sheldrick, 2008 ▸), *SHELXL2018/3* (Sheldrick, 2015 ▸), *shelXle* (Hübschle *et al.*, 2011 ▸), *OLEX2* (Dolomanov *et al.*, 2009 ▸), *publCIF* (Westrip, 2010 ▸).

## full crystallographic data

### Crystal data

**Table:**

C_5_H_9_N_2_^+^·Cl^−^	*D*_x_ = 1.283 Mg m^−^^3^
*M**_r_* = 132.59	Mo *K*α radiation, λ = 0.71073 Å
Orthorhombic, *P*2_1_2_1_2_1_	Cell parameters from 9928 reflections
*a* = 6.3076 (4) Å	θ = 2.8–33.1°
*b* = 9.5490 (6) Å	µ = 0.46 mm^−^^1^
*c* = 11.3951 (8) Å	*T* = 150 K
*V* = 686.34 (8) Å^3^	Prism, colourless
*Z* = 4	0.53 × 0.49 × 0.42 mm
*F*(000) = 280	

### Data collection

**Table:**

Bruker AXS D8 Quest CMOS diffractometer	2499 reflections with *I* > 2σ(*I*)
Detector resolution: 10.4167 pixels mm^-1^	*R*_int_ = 0.029
ω and phi scans	θ_max_ = 33.1°, θ_min_ = 2.8°
Absorption correction: multi-scan (SADABS; Krause *et al.*, 2015)	*h* = −7→9
*T*_min_ = 0.713, *T*_max_ = 0.747	*k* = −14→12
13899 measured reflections	*l* = −15→17
2602 independent reflections	

### Refinement

**Table:**

Refinement on *F*^2^	Hydrogen site location: difference Fourier map
Least-squares matrix: full	All H-atom parameters refined
*R*[*F*^2^ > 2σ(*F*^2^)] = 0.019	*w* = 1/[σ^2^(*F*_o_^2^) + (0.0228*P*)^2^ + 0.0624*P*] where *P* = (*F*_o_^2^ + 2*F*_c_^2^)/3
*wR*(*F*^2^) = 0.050	(Δ/σ)_max_ < 0.001
*S* = 1.11	Δρ_max_ = 0.21 e Å^−^^3^
2602 reflections	Δρ_min_ = −0.14 e Å^−^^3^
111 parameters	Extinction correction: SHELXL-2018/3 (Sheldrick 2015), Fc^*^=kFc[1+0.001xFc^2^λ^3^/sin(2θ)]^-1/4^
0 restraints	Extinction coefficient: 0.034 (6)
Primary atom site location: structure-invariant direct methods	Absolute structure: Refined as an inversion twin
Secondary atom site location: difference Fourier map	Absolute structure parameter: 0.29 (5)

### Special details

**Table:**

Geometry. All esds (except the esd in the dihedral angle between two l.s. planes) are estimated using the full covariance matrix. The cell esds are taken into account individually in the estimation of esds in distances, angles and torsion angles; correlations between esds in cell parameters are only used when they are defined by crystal symmetry. An approximate (isotropic) treatment of cell esds is used for estimating esds involving l.s. planes.
Refinement. Refined as a 2-component inversion twin.

### Fractional atomic coordinates and isotropic or equivalent isotropic displacement parameters (Å^2^)

**Table:**

	*x*	*y*	*z*	*U*_iso_*/*U*_eq_	
Cl1	0.23376 (4)	0.42563 (3)	0.59746 (2)	0.02230 (7)	
N1	0.57372 (15)	0.53197 (10)	0.43072 (8)	0.02210 (18)	
H1	0.481 (3)	0.497 (2)	0.4861 (17)	0.046 (5)*	
N2	0.70984 (13)	0.65758 (9)	0.29255 (7)	0.01820 (16)	
C1	0.75982 (19)	0.46689 (11)	0.39763 (10)	0.0272 (2)	
H1A	0.814 (3)	0.3832 (18)	0.4356 (15)	0.034 (4)*	
C2	0.84515 (17)	0.54576 (12)	0.31046 (10)	0.0244 (2)	
H2	0.975 (3)	0.5379 (16)	0.2678 (15)	0.030 (4)*	
C3	0.54531 (15)	0.64734 (11)	0.36651 (9)	0.01807 (17)	
C4	0.73381 (19)	0.76456 (11)	0.20131 (9)	0.0250 (2)	
H4A	0.872 (3)	0.7478 (19)	0.1651 (14)	0.030 (4)*	
H4B	0.715 (3)	0.8557 (16)	0.2354 (13)	0.029 (4)*	
H4C	0.619 (3)	0.753 (2)	0.1407 (17)	0.043 (5)*	
C5	0.36645 (18)	0.74662 (14)	0.37712 (11)	0.0272 (2)	
H5A	0.296 (3)	0.7479 (19)	0.3043 (18)	0.049 (5)*	
H5B	0.418 (3)	0.834 (2)	0.3930 (19)	0.063 (6)*	
H5C	0.271 (3)	0.713 (2)	0.4340 (17)	0.054 (5)*	

### Atomic displacement parameters (Å^2^)

**Table:**

	*U*^11^	*U*^22^	*U*^33^	*U*^12^	*U*^13^	*U*^23^
Cl1	0.02118 (10)	0.02475 (11)	0.02096 (11)	−0.00241 (8)	0.00199 (8)	0.00210 (8)
N1	0.0232 (4)	0.0225 (4)	0.0205 (4)	−0.0032 (3)	0.0033 (3)	0.0009 (3)
N2	0.0171 (4)	0.0185 (3)	0.0191 (3)	−0.0025 (3)	0.0016 (3)	−0.0015 (3)
C1	0.0284 (5)	0.0239 (4)	0.0294 (5)	0.0040 (4)	0.0037 (5)	0.0045 (4)
C2	0.0204 (4)	0.0250 (5)	0.0277 (5)	0.0025 (4)	0.0038 (4)	0.0007 (4)
C3	0.0170 (4)	0.0199 (4)	0.0173 (4)	−0.0033 (3)	0.0009 (3)	−0.0035 (3)
C4	0.0286 (5)	0.0219 (4)	0.0245 (4)	−0.0042 (4)	0.0042 (4)	0.0045 (4)
C5	0.0228 (4)	0.0294 (5)	0.0295 (6)	0.0044 (4)	0.0033 (4)	−0.0041 (4)

### Geometric parameters (Å, º)

**Table:**

N1—H1	0.923 (19)	C2—H2	0.955 (17)
N1—C1	1.3806 (14)	C3—C5	1.4786 (15)
N1—C3	1.3346 (14)	C4—H4A	0.980 (17)
N2—C2	1.3821 (14)	C4—H4B	0.960 (15)
N2—C3	1.3405 (12)	C4—H4C	1.005 (19)
N2—C4	1.4654 (13)	C5—H5A	0.94 (2)
C1—H1A	0.971 (17)	C5—H5B	0.92 (2)
C1—C2	1.3578 (16)	C5—H5C	0.94 (2)
			
C1—N1—H1	124.4 (11)	N2—C3—C5	126.54 (10)
C3—N1—H1	125.8 (11)	N2—C4—H4A	106.1 (10)
C3—N1—C1	109.63 (9)	N2—C4—H4B	109.4 (9)
C2—N2—C4	125.43 (9)	N2—C4—H4C	109.6 (11)
C3—N2—C2	109.20 (9)	H4A—C4—H4B	115.3 (15)
C3—N2—C4	125.23 (9)	H4A—C4—H4C	109.4 (13)
N1—C1—H1A	123.3 (10)	H4B—C4—H4C	107.0 (15)
C2—C1—N1	106.69 (10)	C3—C5—H5A	107.2 (12)
C2—C1—H1A	129.9 (10)	C3—C5—H5B	109.3 (14)
N2—C2—H2	121.0 (10)	C3—C5—H5C	108.9 (12)
C1—C2—N2	106.96 (10)	H5A—C5—H5B	109.3 (18)
C1—C2—H2	131.9 (10)	H5A—C5—H5C	108.1 (16)
N1—C3—N2	107.52 (9)	H5B—C5—H5C	113.8 (18)
N1—C3—C5	125.94 (10)		
			
N1—C1—C2—N2	0.25 (13)	C3—N1—C1—C2	−0.20 (13)
C1—N1—C3—N2	0.06 (12)	C3—N2—C2—C1	−0.22 (12)
C1—N1—C3—C5	−178.91 (10)	C4—N2—C2—C1	−175.97 (10)
C2—N2—C3—N1	0.10 (11)	C4—N2—C3—N1	175.86 (9)
C2—N2—C3—C5	179.06 (10)	C4—N2—C3—C5	−5.17 (16)

### Hydrogen-bond geometry (Å, º)

**Table:**

*D*—H···*A*	*D*—H	H···*A*	*D*···*A*	*D*—H···*A*
N1—H1···Cl1	0.923 (19)	2.122 (19)	3.0396 (10)	172.3 (17)
C2—H2···Cl1^i^	0.955 (17)	2.695 (17)	3.6084 (11)	160.2 (13)
C4—H4*C*···Cl1^ii^	1.005 (19)	2.85 (2)	3.6601 (12)	138.3 (14)
C5—H5*A*···Cl1^ii^	0.94 (2)	2.887 (19)	3.6415 (13)	138.0 (14)

Symmetry codes: (i) −*x*+3/2, −*y*+1, *z*−1/2; (ii) −*x*+1/2, −*y*+1, *z*−1/2.
